# Health Tract, No. 24

**Published:** 1862-01

**Authors:** 


### HEALTH TRACT, No. 24.

### SOUR STOMACH.

Nature provides a liquid (the gastric juice) in the stomach, sufficient to dissolve as much food as the system requires, and no more. Whatever is eaten beyond what is needed has no gastric juice to dissolve it, and being kept at the temperature of the stomach, which is about a hundred degrees, it begins to decompose—that is, to sour—in one, two, three, or more hours, just as new cider begins to sour in a few hours. In the process of souring, gas is generated as in the cider-barrel, the bung is thrown out, and some of the contents run over at the bung-hole, because in souring, the contents expand, and require more room. So with the stomach. It may be but partially filled by a meal; but if more has been swallowed than wise nature has provided gastric juice for, it begins to sour, to ferment, to distend, and the man feels uncomfortably full. He wants to belch. That gives some relief. But the fermentation going on, he gets the "belly ache" of childhood or some other discomfort, which lasts for several hours, when nature succeeds in getting rid of the surplus, and the machinery runs smoothly again. But if these things are frequently repeated, the machinery fails to rectify itself, looses the power of reädjustment, works with a clog, and the man is a miserable dyspeptic for the remainder of life; and all from his not having had wit enough to know when he had eaten a plenty, and being foolish enough, when he had felt the ill effects of thus eating too much, to repeat the process an indefinite number of times; and all for the trifling object of feeling good for the brief period of its passing down the throat. For each minute of that good he pays the penalty of a month of such suffering as only a dyspeptic can appreciate. What a fool man is! He is a numskull, a goose, a sheep, a goat, a jackass.

